# Fibromatose desmoïde du sein: à propos d'un cas et une revue de la literature

**DOI:** 10.11604/pamj.2015.21.88.7124

**Published:** 2015-06-03

**Authors:** Sarah Amourak, Fatimazahra Fdili Alaoui, Sofia Jayi, Hikmat Chaara, Moulay Abdelilah Melhouf

**Affiliations:** 1 Université Sidi Mohammed Benabdellah, Service de Gynécologie-Obstétrique 2, CHU Hassan II de Fès, Maroc

**Keywords:** Fibromatose desmoide, sein, récidive, marge saine, desmoid fibromatosis, sein, relapse, healthy margin

## Abstract

La fibromatose desmoïde mammaire est une entité rare, mimant sur le plan clinique et échographique un cancer du sein. Seule l'histologie apportera le diagnostic en objectivant une prolifération de cellule fusiforme (fibro et myofibroblastique sans atypies nucléaires), agencée en faisceaux, mêlés à des bandes de collagène, sans composante épithéliale. Le diagnostic différentiel se pose essentiellement avec le carcinome métaplasique à cellules fusiformes. L’évolution est strictement locale, avec un grand pouvoir récidivant. L'exérèse chirurgicale complète avec des marges saines (jusqu’à 3cm) est le traitement de choix, la radiothérapie reste une option thérapeutique en complément de la chirurgie dans les exérèses incomplètes et en cas de récidives multiples. A travers notre cas et une revue de la littérature, nous essayerons de mettre le point sur le diagnostic de cette entité rare et de sa prise en charge puisqu'elle va conditionner le pronostic.

## Introduction

Décrites pour la première fois en 1832, la fibromatose desmoïde est une tumeur bénigne, mésenchymateuses, développées à partir des structures musculo-aponévrotiques. La localisation mammaire est très rare, certains auteurs la considère comme un sous-groupe des sarcomes des tissus mous et ne font pas de différence entre une fibromatose agressive et un fibrosarcome de bas grade.

## Patient et observation

**Figure 1:**
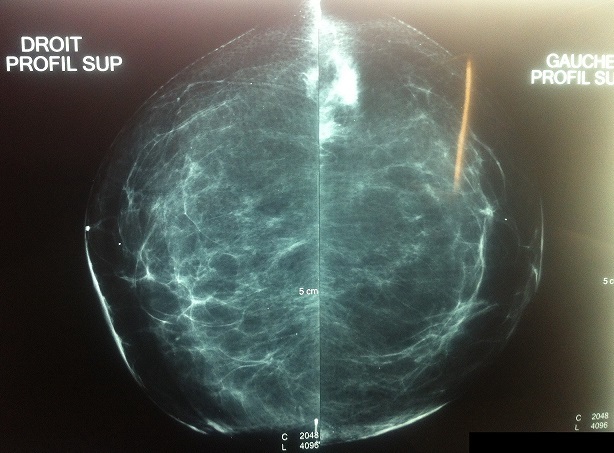
Lésions suspectes mimant un cancer du sein

Patiente âgée de 30 ans, célibataire, ayant comme ATCD une tumerectomie il y a 1 an (tumeur de la paroi thoracique droite) dont le résultat anatomopathologique est en faveur d'une fibromatose de la paroi thoracique. L'examen trouve une tumeur du QSE du sein droit, faisant 3/2cm, douloureuse, ferme et une autre tumeur du QSE du sein gauche, faisant 2/1,5cm, sans rétraction ni écoulement mamelonnaire. L’échographie mammaire + mammographie: (1) aspect évocateur d'une lésion maligne du QSE du sein gauche c'est une masse bilobée à contours irréguliers spiculés classée ACR5; (2) aspect évocateur d'une lésion suspecte du QSE du sein Droit classée ACR4. La micro-biopsie a révélé un aspect histologique pouvant évoquer une fibromatose desmoïde sans être exclusif, nous avons complété par une étude immuno-histo-chimique en faveur d'une fibromatose desmoïde. Elle a bénéficié d'une double tumerectomie avec des marges saines de 3cm (Figure 1).

## Discussion

Les tumeurs desmoïde mammaires sont très rares, environ 100 cas ont été rapportés dans la littérature médicale à ce jour. La fréquence rapportée par les différents auteurs, des tumeurs desmoïde mammaires, quelque soit le sexe ne dépasse pas 3.5% des tumeurs fibreuses, et 0.2% de l'ensemble des tumeurs du sein [1–3]. La localisation mammaire est rencontrée à tout âge, avec maximum entre 20 et 40 ans, ce qui correspond à l’âge de notre patiente. Cette fibromatose desmoïde mammaire peut se voir aussi chez l'homme. La physiopathologie constitue le côté obscure de cette pathologie, elle pourrait résulter de la sommation de ces 3 facteurs: (1) initiation de la prolifération cellulaire suite à un traumatisme, Des cas sont décrits après traumatisme, ou après chirurgie du sein comme pour les chirurgies de réduction mammaire [1, 4, 5], ou après prothèse mammaire [6, 7]; (2) un effet promoteur des hormones sexuelles: plusieurs arguments plaident en faveur de cet effet en plus de l´hormonodépendance des tumeurs desmoïdes, puisque les fibromatoses surviennent préférentiellement chez les multipares, au cours de la grossesse, dans un contexte d´hyperoestrogénie ou lors de la prise d´une contraception orale [8, 9]; (3) un terrain génétique par trouble de la régulation de la croissance des fibroblastes.

La survenue d'une fibromatose pourrait être le témoin d'une anomalie génétique familiale. En effet, ont été décrit des cas de fibromatoses familiales multicentriques isolées; ou entrant dans le cadre d'un syndrome de GARDNER associant une polypose colique, des malformations osseuses, des kystes épidermoides et des tumeurs des tissus mous dont les tumeurs desmoïde. En 1986, REITAMO avait défini le syndrome desmoïde comme une entité associant la fibromatose à des anomalies du squelette (exostoses, géodes; sur densités au niveau des fémurs et des mandibules; sacralisation de la 5^ème^ vertèbre lombaire). Ce syndrome serait autosomique dominant à pénétrance variable [10].

Cliniquement, cette tumeur se manifeste comme une masse, ferme, de taille variable, indolore le plus souvent périphérique, pouvant être associée à un épaississement cutané ou rétraction mamelonnaire. Radiologiquement, on peut avoir à la mammographie une masse spiculée non calcifié, les micro ou les macrocalcifications sont rares, par contre dans le 1/3 des cas aucune lésion n'est objectivée à la mammographie. L'aspect échographique correspond à une masse solide spiculée ou microlobulée irrégulière, hypoéchogène, on peut même objectiver une atteinte des muscles pectoraux ou intercostaux, donc cette lésion mime sur le plan clinique et radiologique une lésion maligne. L'IRM mammaire n'a aucun intérêt à visée diagnostique par contre elle peut évaluer l'extension tumorale des volumineuses lésions en objectivant une masse à contours mal limités, spiculés iso intense aux muscles, en iso/hypersignal T2 d'intensité variable et hétérogène [11].

Sur le plan histologique c'est une lésion mal limitée, de forme étoilée, ferme et blanchâtre mimant macroscopiquement un néo. L'examen microscopique objective une prolifération de cellule fusiforme (fibro et myofibroblastique sans atypies nucléaires), agencée en faisceaux, mêlés à des bandes de collagène, sans composante épithéliale associée. En immuno-histochimie, les cellules expriment de façon intense et diffuse l'actine musculaire lisse alors la desmine n'est exprimée que par de rares cellules (profil myofibroblastique). Il existe très fréquemment une positivité pour la bêta-caténine qui est de façon caractéristique localisée au niveau de noyaux. Les récepteurs estrogènes et à la progestérone ne sont pas détectables par immunohistochimie dans la fibromatose mammaire, comme son homologue extramammaire, alors que pour celui-ci des réponses thérapeutiques à une hormonothérapie ont été rapportées.

Le diagnostic différentiel se pose essentiellement avec le carcinome métaplasique à cellules fusiformes car l'expression caractéristique pour la cytokératine est parfois focale, détectée sur la pièce de résection ce qui va confirmer le diagnostic, Il se pose également devant une tumeur phyllode d'agressivité intermédiaire ou maligne, devant le fibrosarcome ou le carcinome myoépithéliale ou encor devant une cicatrice fibreuse car ce sont les mêmes cellules fusiformes mais c'est le contingent inflammatoire présent en cas de cicatrice fibreuse qui va faire la différence. La particularité de cette fibromatose quelque soit le siège c'est l'infiltration du tissu adjacent

Le traitement repose essentiellement sur l'exérèse chirurgicale complète avec des marges saines (jusqu’à 3cm) [12, 13]. Les récidives sont fréquentes de 18 à 29% (3 à 6 ans), l'atteinte thoracique des muscles et des côtes est possible. Chez certaines femmes, une mastectomie est recommandée en cas de récidives multiples, en cas d'une tumeur de grand volume ou en cas de difficulté du diagnostic histologique. La place de la radiothérapie est très controversée dans la littérature, son efficacité est dose dépendante, ainsi que le contrôle tumoral est de 60 à 80% pour une dose totale administrée de 50 à 60 GY. Les autres traitements adjuvants ont été aussi essayés: anti inflammatoire, anti œstrogène et une chimiothérapie à faible dose.

## Conclusion

La fibromatose mammaire est une entité rare, mimant sur le plan clinique et radiologique un cancer du sein. Seule l'histologie apportera le diagnostic. Histologiquement elle correspond à une prolifération fibroblastique bénigne, d’évolution strictement locale, avec un grand pouvoir récidivant. L'exérèse complète avec des marges saines de sécurité constitue le traitement de choix.
